# Comparison of Airway-Based and Transpulmonary Mechanical Power Across Ventilation Conditions in Moderate-to-Severe ARDS

**DOI:** 10.3390/life16101662

**Published:** 2026-10-03

**Authors:** Arie Soroksky, Alina Lianskaya, Ronen Balmor Gingy, Nugzar Riziny, Adam Li Goldstein, Emad Tayem, Ori Galante, Asaf Miller, Milena Tocut

**Affiliations:** 1 Intensive Care Department, E. Wolfson Medical Center, Holon 58100, Israel; aries@wmc.gov.il (A.S.); gingyb@wmg.gov.il (R.B.G.); emadt@wmc.gov.il (E.T.); 2 Gray Faculty of Medicine and Health Sciences, Tel Aviv University, Tel Aviv 6139001, Israel; alinali@wmc.gov.il (A.L.); nogzar@wmc.gov.il (N.R.); adamleeg@wmc.gov.il (A.L.G.); 3 Anesthesia Department, E. Wolfson Medical Center, Holon 58100, Israel; 4 Surgical Department A Trauma Division, E. Wolfson Medical Center, Holon 58100, Israel; 5 Faculty of Health Sciences, Ben-Gurion University of the Negev, Be’er Sheva 8410501, Israel; ori.galente@gmail.com; 6 Medical Intensive Care Unit, Soroka University Medical Center, Be’er Sheva 8410501, Israel; 7 Rappaport Faculty of Medicine, Technion-Israel Institute of Technology, Haifa 3200003, Israel; asaf.miller@gmail.com; 8 General Intensive Care Unit, Rambam Health Care Campus, Haifa 3109601, Israel; 9 Internal Medicine C Department, Wolfson Medical Center, 61 Halochamim Street, Holon 58100, Israel

**Keywords:** mechanical ventilation, acute respiratory distress syndrome (ARDS), ventilator-induced lung injury (VILI), mechanical power, transpulmonary mechanical power

## Abstract

**Background:** Mechanical ventilation is essential for managing acute respiratory distress syndrome (ARDS) but may contribute to ventilator-induced lung injury (VILI). Mechanical power (MP), an integrative measure combining tidal volume, respiratory rate, and airway pressures, has emerged as a predictor of VILI. However, conventional MP calculations based on airway driving pressure (ΔP) reflect the mechanical load applied to the entire respiratory system, including both lung parenchyma and chest wall. Estimation of transpulmonary pressure using esophageal pressure allows calculation of transpulmonary mechanical power (TPMP), which may more accurately represent lung stress. **Methods:** We conducted a retrospective observational study of 20 mechanically ventilated ICU patients with available esophageal pressure measurements at Wolfson Medical Center between 2012 and 2015. MP and TPMP were calculated across ventilation modes, including continuous mandatory ventilation assist/control (CMV/AC), synchronized intermittent mandatory ventilation (SIMV), and continuous positive airway pressure/pressure support ventilation (CPAP/PSV). Paired comparisons were analyzed using the Wilcoxon signed-rank test; repeated-measures Friedman tests were used to assess within-patient differences in MP, TPMP, and the MP−TPMP difference across the four ventilation conditions. **Results:** At baseline, MP and TPMP values were similar, with only a small absolute difference despite statistical significance (median 20 vs. 20 J/min; *p* = 0.0107). Differences were more pronounced and mode-dependent during CMV/AC, CPAP/PSV, and SIMV. During CMV/AC, TPMP exceeded MP (median 23 vs. 22 J/min; *p* = 0.0016). In contrast, TPMP was lower than MP during CPAP/PSV (median 14 vs. 20 J/min; *p* < 0.0001) and SIMV (median 17 vs. 23 J/min; *p* = 0.0073). In a repeated-measures analysis, the within-patient MP−TPMP difference varied significantly across ventilation conditions (χ^2^ = 33.06, df = 3, *p* < 0.0001), while MP alone did not differ significantly across conditions (χ^2^ = 6.90, df = 3, *p* = 0.075); these findings represent associations with ventilation condition and clinical state rather than a causal effect of ventilation mode. **Conclusions:** This exploratory study demonstrates that airway-based MP and esophageal pressure-derived TPMP provide different estimates of mechanical load and that the magnitude and direction of these differences vary across ventilation conditions. These findings support the physiological relevance of considering transpulmonary pressure when characterizing respiratory mechanical load. However, this study does not establish that TPMP-guided ventilation improves clinical outcomes or reduces VILI. The potential clinical value of TPMP should therefore be evaluated in larger prospective studies.

## 1. Introduction

Mechanical ventilation is an essential intervention for acute respiratory failure, particularly in patients with acute respiratory distress syndrome (ARDS) [1]. However, it can result in ventilator-induced lung injury (VILI) due to high pressures and volumes [2,3]. To balance adequate ventilation with lung protection, indices such as mechanical power (MP) and transpulmonary mechanical power (TPMP) have been developed. MP quantifies the amount of energy per minute transferred from the ventilator to the respiratory system and is an integrative index that includes tidal volume (TV), respiratory rate (RR), airway pressures including Ppeak, Pplat, positive end-expiratory pressure (PEEP), airway resistance, and lung compliance [4,5,6].

MP is considered a central predictor of VILI, rather than pressure or volume thresholds alone. The dynamic combination of all energy components is now understood to have a greater impact than any single variable, as it reflects the cumulative load on lung tissue over time [7]. Studies have demonstrated that high MP is associated with an increased risk of VILI and mortality due to lung over-distension and repetitive pressures [8,9]. Therefore, reducing VILI risk requires a deep understanding of the interaction between the MP parameters, sedation status, and the patient’s breathing effort.

Calculating MP based on airway driving pressure (ΔP) reflects the load on the entire respiratory system, comprising the lung and chest wall [10]. In conditions of increased chest wall load, this may overestimate the load on the lung parenchyma [11]. Estimation of pleural pressure using esophageal pressure enables calculation of transpulmonary pressure and may provide complementary information regarding the mechanical load applied across the lung, beyond that obtained from airway pressure alone [12]. This technique has been shown to improve oxygenation and compliance in patients with moderate or severe ARDS [13].

Esophageal pressure is commonly used as a surrogate estimate of pleural pressure, allowing calculation of transpulmonary pressure and providing additional information regarding lung-specific mechanical load. However, esophageal pressure does not represent a direct or spatially uniform measurement of pleural pressure. Its accuracy and interpretation may be influenced by catheter position, balloon filling volume, calibration technique, patient position, mediastinal effects, and regional variations in pleural pressure. Therefore, esophageal pressure-derived transpulmonary pressure and TPMP should be interpreted within these methodological constraints [12,13].

Sedation depth directly affects respiratory system behavior: patients under deep sedation with neuromuscular blockade exert minimal breathing effort, an approach that has been shown to improve survival in moderate-to-severe ARDS by eliminating patient–ventilator asynchrony [14]. Conversely, patients in partial wakefulness initiate spontaneous breaths that may generate significant negative pleural pressures that add to the strain applied by the MP [15]. This additional load, often described as patient self-inflicted lung injury, is not measured by the MP parameters alone [16]. Furthermore, strong spontaneous effort can cause pendelluft, the movement of gas from nondependent to dependent lung regions, causing occult regional overstretch even when TV appears low [17,18].

Conventional MP has been increasingly investigated as an integrated marker of ventilator-associated mechanical load. Thus, it remains unclear whether airway-based MP accurately reflects the mechanical load applied specifically to the lung parenchyma across different modes of ventilation. This distinction may be particularly important during transitions from controlled to partially supported or spontaneous breathing, when changes in chest-wall mechanics and patient respiratory effort may substantially alter pleural and transpulmonary pressures without being adequately captured by airway pressure alone. Consequently, the relationship between conventional MP and TPMP may vary according to ventilation mode, potentially affecting the interpretation of lung stress and ventilatory management. However, direct within-patient comparisons of MP and TPMP across controlled, partially supported, and spontaneous ventilation modes remain limited. Therefore, the present study compared airway-based MP with esophageal pressure-derived TPMP across CMV/AC, SIMV, and CPAP/PSV. We aimed to determine whether the magnitude and direction of the difference between MP and TPMP vary according to ventilation mode and to explore the potential implications of these differences for individualized assessment of mechanical load in patients with moderate or severe ARDS.

## 2. Methods

This was a retrospective observational study utilizing data from the medical records of 20 ventilated patients admitted to the intensive care unit (ICU) at Wolfson Medical Center in Israel between 2012 and 2015. The study was approved by the medical center’s Helsinki Committee (approval number: 0019-25-WOMC). Informed consent was waived because of the retrospective design.

Inclusion criteria included age > 18 years, MP via endotracheal tube, availability of esophageal pressure, Paw, VT, RR data, and documentation of the ventilation mode. Exclusion criteria included previous lung/chest/esophageal surgery, esophageal pathology, presence of chest tube, kyphoscoliosis, incomplete records, or hemodynamic instability during measurements. Collected data included pressures (PEEP, Ppeak, Pplat, ΔP, Cdyn), esophageal pressure, TV (liters), and RR. TPMP and MP indices were calculated according to accepted equations. Different depths of sedation were achieved and were guided by the Richmond Agitation–Sedation Scale (RASS) score, adjusted according to the ventilation condition under study.

Measurements were obtained sequentially for each patient within a single predefined physiological protocol session. Following baseline assessment, patients were transitioned through controlled mechanical ventilation (CMV/AC), synchronized intermittent mandatory ventilation (SIMV), and continuous positive airway pressure/pressure support ventilation (CPAP/PSV), with each condition maintained for approximately 60 min of stabilization prior to data acquisition so that respiratory mechanics and hemodynamic status were reasonably stable at the time of each measurement. All four conditions were therefore assessed within the same clinical encounter rather than at different points in the patients’ broader clinical course.

During CPAP/PSV, patients received a median PEEP/CPAP level of 12 cm H_2_O (IQR 10.0–15.0; range 6–25) with a median inspiratory pressure support level of 16 cm H_2_O (IQR 13.8–18.0; range 10–24) above PEEP, as detailed in Supplementary Table S1.

The esophageal balloon technique used a standard Nutrivent multifunctional nasogastric catheter (SIDAM Group, Medical Solutions). The balloon was inflated with 0.5 to 1.0 mL of air, and measurements were obtained with patients in a semi-recumbent position (head of bed elevated approximately 30°). Before each set of measurements, correct balloon catheter position was verified using the dynamic occlusion test, with an esophageal-to-airway pressure change ratio (ΔPes/ΔPaw) close to 1.0 and the presence of cardiac oscillation artifact on the pressure tracing taken as additional confirmation of correct positioning. These procedures were verified against our ICU’s standard operating protocols and source records from the study period. Tracings lacking cardiac oscillation artifact or failing the occlusion test were excluded, and, where feasible, the measurement was repeated.

ARDS was defined by the accepted Berlin criteria, which require all of the following:

1. Timing: respiratory symptoms begin within 1 week of a known clinical insult (e.g., sepsis, pneumonia, aspiration, trauma) or new/worsening respiratory symptoms.
2. Chest imaging: bilateral opacities on chest X-ray or CT scan and opacities that are not fully explained by pleural effusions, lobar/lung collapse, or pulmonary nodules.
3. Origin of edema: respiratory failure is not fully explained by cardiac failure or fluid overload, and if there is no obvious ARDS risk factor, an objective assessment (e.g., echocardiography) may be needed to exclude hydrostatic pulmonary edema.
4. Oxygenation impairment: the patient must be receiving PEEP or CPAP ≥ 5 cm H_2_O, and severity is classified by the PaO_2_/FiO_2_ (P/F) ratio: mild ARDS is defined by a P/F ratio >200 to ≤300 mmHg, moderate ARDS by >100 to ≤200 mmHg, and severe ARDS by ≤100 mmHg.

### 2.1. Calculation Methods

Equation used for MP:

MP (J/min) = 0.098 × RR × V_T_ × (Ppeak − ΔP/2)

where ΔP = Pplat − PEEP.

Substituting the transpulmonary driving pressure (ΔPtp) for airway driving pressure (ΔP) in the MP equation provides an alternative estimate of mechanical load that incorporates esophageal pressure-derived changes in pleural pressure. Conventional ΔP reflects the pressure applied to the respiratory system as a whole, including both the lung and chest wall. In contrast, transpulmonary pressure, calculated as the difference between airway pressure and estimated pleural pressure, provides additional information regarding the pressure developed across the lung. However, because esophageal pressure is an indirect estimate of pleural pressure and the TPMP equation used in this study represents an adaptation of a conventional ventilator-derived MP equation, TPMP should be interpreted as an estimate of transpulmonary mechanical load rather than a direct measurement of lung parenchymal stress or total lung MP.

The equation used for TPMP, based on transpulmonary pressure:

TPMP (J/min) = 0.098 × RR × V_T_ × (Ppeak − ΔPtp/2)

where ΔPtp = Ptp,_end-insp_ − Ptp,_end-exp_.

MP, expressed in joules per minute (J/min), was grouped a priori into descriptive numerical ranges for visualization purposes, rather than into clinically validated risk categories. For descriptive visualization, MP and TPMP values were grouped into predefined mechanical-power ranges of <12, 12–17, >17 to <22, and ≥22 J/min. These ranges were used solely to facilitate visualization and comparison of the distribution of MP and TPMP values across ventilation conditions. They should not be interpreted as validated clinical thresholds for VILI, prognosis, or therapeutic decision-making. In particular, clinically validated thresholds for TPMP have not been established, and conventional airway-based MP thresholds cannot necessarily be extrapolated to TPMP.

**Figure 1:**
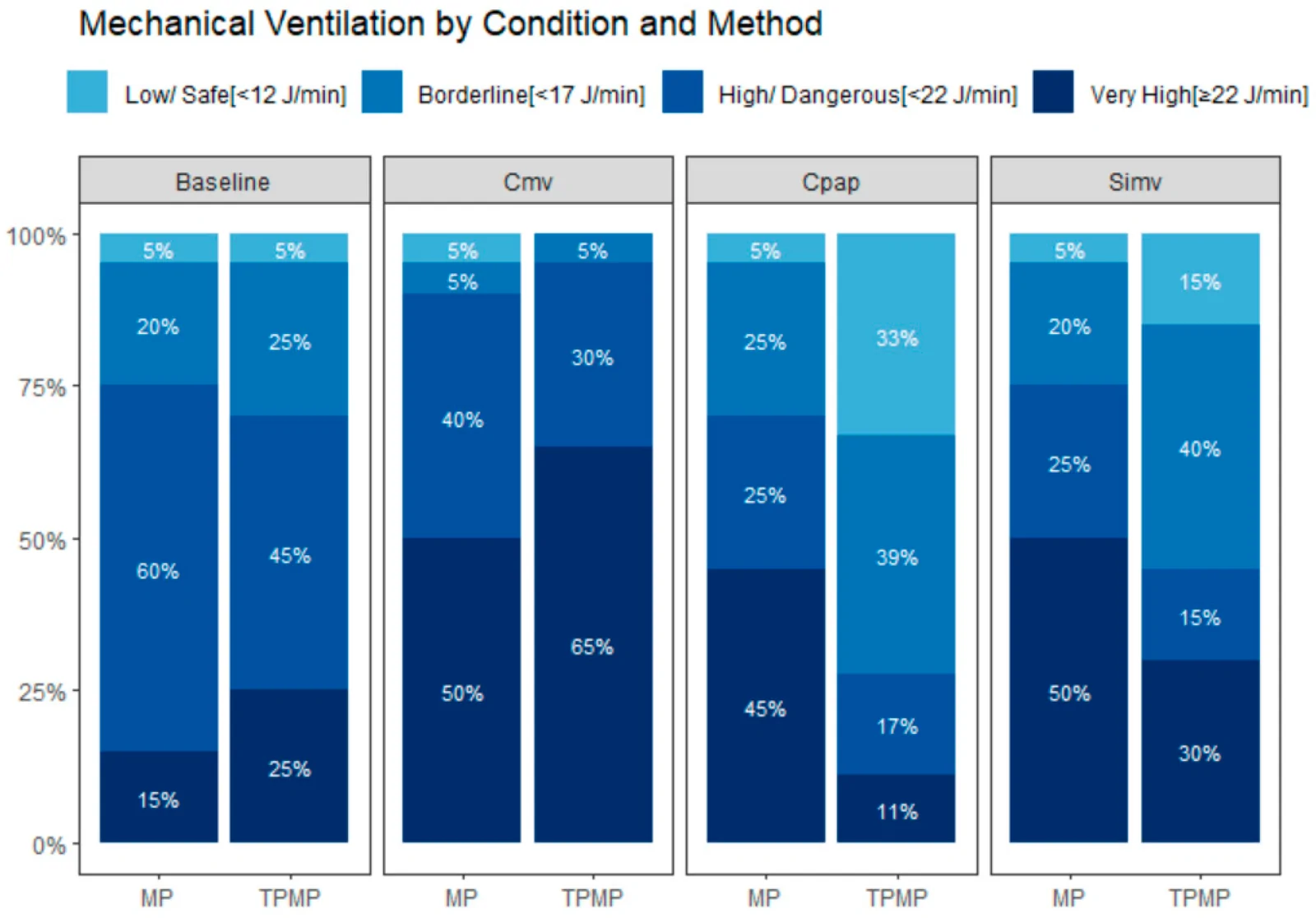
Distribution of conventional mechanical power (MP) and transpulmonary mechanical power (TPMP) across predefined mechanical-power ranges under the evaluated ventilation conditions. Bars represent the proportion of measurements falling within each predefined MP range (<12, 12–17, >17 to <22, and ≥22 J/min) for conventional MP and TPMP across baseline, CMV/AC, CPAP/PSV, and SIMV conditions. These categories are presented descriptively to facilitate comparison of the distributions and should not be interpreted as validated clinical risk thresholds for TPMP. MP, mechanical power; TPMP, transpulmonary mechanical power; CMV/AC, controlled mechanical ventilation/assist control; CPAP/PSV, continuous positive airway pressure/pressure support ventilation; SIMV, synchronized intermittent mandatory ventilation.

For descriptive purposes, these four ranges were labeled Tier 1 (<12 J/min), Tier 2 (12–17 J/min), Tier 3 (>17 to <22 J/min), and Tier 4 (≥22 J/min) (Figure 1). These tier labels are purely descriptive and numerical; they do not represent validated categories of safety, danger, or risk, and they should not be used to guide ventilatory management or interpreted as thresholds prompting a specific therapeutic action.

Figure 1 illustrates the distribution of mechanical power and transpulmonary mechanical power across predefined mechanical-power ranges according to ventilation condition.

### 2.2. Statistical Analysis

Mechanical ventilation methods were compared within subjects based on two paired measures: MP and TPMP. Due to the small sample size and non-normal distribution of continuous variables, these were summarized using medians and interquartile ranges (IQR, Q1–Q3).

Categorical variables were summarized using counts and percentages. Paired comparisons between MP and TPMP values were performed using the Wilcoxon signed-rank test (paired Wilcoxon test). The MP and TPMP tier categories (Tier 1 [<12 J/min], Tier 2 [12–17 J/min], Tier 3 [>17 to <22 J/min], and Tier 4 [≥22 J/min]) were visualized using stacked bar charts, with the proportion of measurements in each tier reported separately by ventilation condition. Because repeated measurements were obtained from the same patients across ventilation conditions, we additionally performed Friedman tests (a nonparametric repeated-measures analogue of ANOVA) on MP, on TPMP, and on the within-patient MP−TPMP difference across the four conditions, using the patients with complete data for all conditions (n = 20). Statistical significance was defined as a two-sided Type I error rate of 0.05. All statistical analyses were conducted using the R Core Team (2025). R: A Language and Environment for Statistical Computing (R Foundation for Statistical Computing, Vienna, Austria).

## 3. Results

The cohort comprised 20 patients with a median age of 72.5 years (IQR 57.8–79.0). The majority of patients were male (75%), while females accounted for 25% of the cohort. Body mass index (BMI) data were available for 18 patients, with a median BMI of 24.6 kg/m^2^ (IQR 22.9–27.6).

The median length of hospital stay was 43.0 days (IQR 17.2–61.2). Patients required prolonged ICU management, with a median ICU length of stay of 14.0 days (IQR 9.8–29.8).

Disease severity on admission was high, reflected by a median APACHE II score of 25.5 (IQR 17.0–27.0). Correspondingly, the median APACHE II-predicted in-hospital mortality was 54.8% (IQR 27.1–63.0). Observed in-hospital mortality was 50%, with 10 patients dying during hospitalization.

The burden of organ dysfunction was considerable, with a median of 5.0 (IQR 4.0–5.0) failing organs per patient. Nearly all patients experienced shock during their ICU course (95%). Acute kidney injury (AKI) occurred in 75% of patients. Additional organ involvement included liver dysfunction in 35%, hematologic failure in 45%, and central nervous system involvement in 25% of cases.

ARDS was highly prevalent and was present at the time of intubation in 90% of patients and on ICU admission in 80% of patients. During the ICU stay, 30% of patients developed ARDS, reflecting dynamic respiratory trajectories within the cohort.

Pre-existing comorbidities were common, with underlying heart disease, lung disease, and chronic kidney disease each present in 30% of patients. Most admissions were medical in nature (90%), with surgical admissions accounting for 10%. Pneumonia was the predominant diagnosis on admission (75%), followed by sepsis and abdominal sepsis. Among non-survivors, pneumonia (56%) and sepsis (44%) were the leading direct causes of death.

Organ support-free days were limited, consistent with severe critical illness. The median number of ventilator-free days after 28 days was 1.0 (IQR 0.0–4.0). AKI-free days were similarly low, with a median of 1.5 days (IQR 0.0–7.5). Median renal replacement therapy-free days were 3.5 (IQR 0.0–10.25), while vasopressor-free days had a median of 5.0 (IQR 1.0–11.75).

Ventilator-associated pneumonia developed in 7 patients (35%). Among affected patients, the median time from initiation of MP to VAP onset was 9.0 days (IQR 5.5–15.0).

Overall, 9 patients (45%) were successfully extubated during hospitalization, while 11 patients (55%) remained intubated or died prior to extubation. Extubation beyond 48 h occurred in 8 patients (40%), whereas 12 patients (60%) were not extubated after this time point. Reintubation after 48 h was required in 4 patients (20%). Tracheostomy was performed in 6 patients (30%), indicating prolonged ventilatory dependence in a substantial proportion of the cohort (Table 1).

MP and TPMP were evaluated across baseline conditions and different modes of mechanical ventilation, including CMV/AC, CPAP/PSV, and SIMV. Ventilator settings and sedation depth for each condition are summarized in Supplementary Table S1. At baseline, 20 paired measurement sets were analyzed (20 patients). Median values of MP and TPMP were similar at 20 J/min, and the ranges overlapped substantially. Despite these similarities, a statistically significant difference between MP and TPMP was observed (*p* = 0.0107).

In a repeated-measures analysis accounting for the within-patient structure (Friedman test, n = 20 patients with complete data for all conditions), TPMP differed significantly across ventilation conditions (χ^2^ = 25.84, df = 3, *p* < 0.0001), and the within-patient MP−TPMP difference differed significantly across conditions (χ^2^ = 33.06, df = 3, *p* < 0.0001), whereas MP alone did not reach statistical significance across conditions (χ^2^ = 6.90, df = 3, *p* = 0.075).

**Figure 2:**
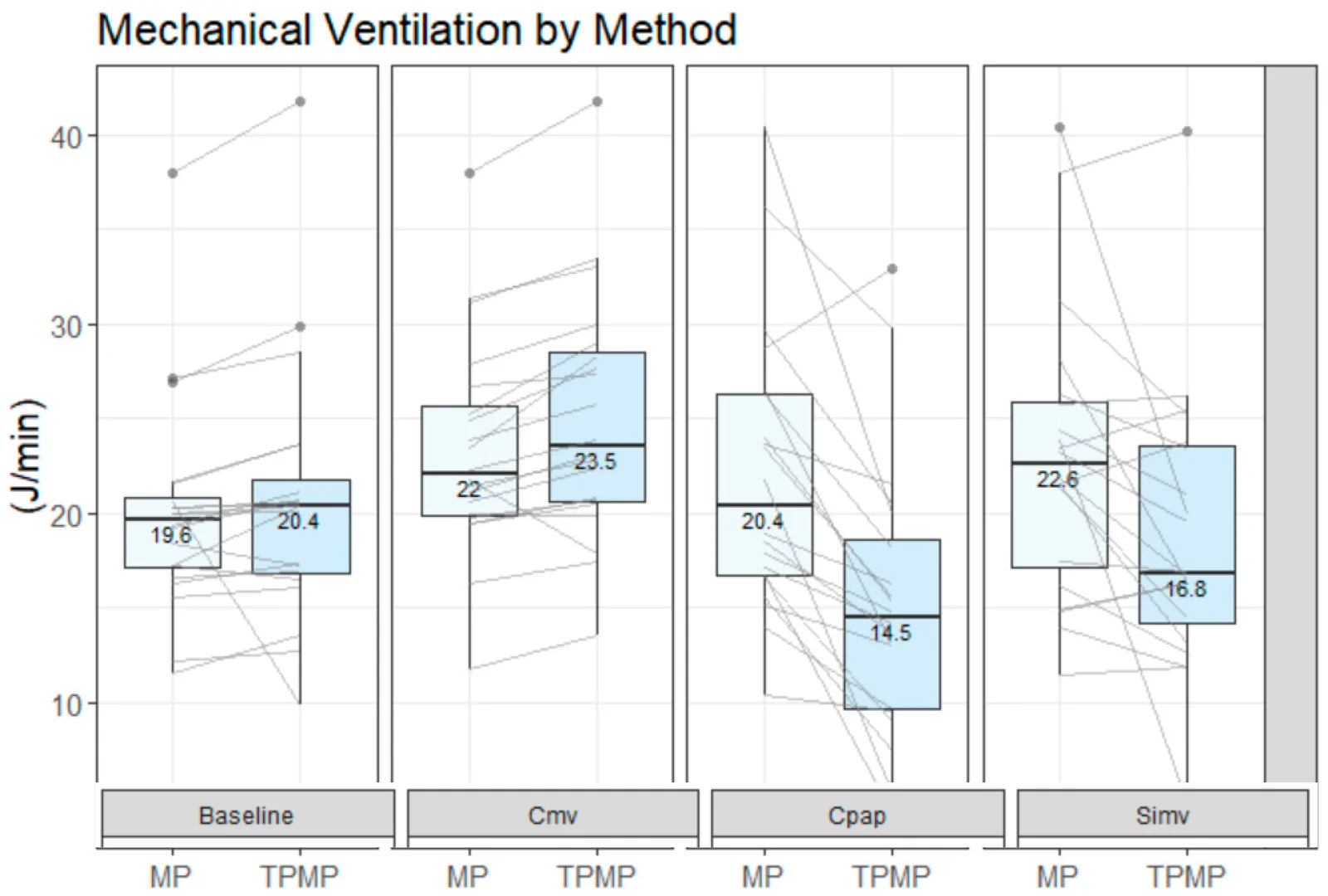
Individual patient-level comparisons of mechanical power (MP) and transpulmonary mechanical power (TPMP) across ventilation modes. Each pair of connected points represents measurements obtained from the same patient, allowing visualization of within-patient differences and interindividual variability. Panels show baseline, controlled mechanical ventilation (CMV/AC), continuous positive airway pressure/pressure support ventilation (CPAP/PSV), and synchronized intermittent mandatory ventilation (SIMV). Only complete paired observations are displayed for each comparison. MP and TPMP are expressed in J/min.

Individual paired MP and TPMP values are presented in Figure 2. Patient-level visualization demonstrated substantial interindividual variability in both measures. The paired plots also illustrate how the within-patient relationship between MP and TPMP varied according to ventilation mode, complementing the group-level comparisons. Given the small cohort, these individual trajectories provide additional information regarding the distribution and consistency of the observed differences and allow assessment of the extent to which the group-level findings were representative of individual patients.

Figure 2 illustrates the individual patient-level comparison of conventional and transpulmonary mechanical power according to ventilation mode.

During CMV/AC mode, 20 paired measurement sets were available (20 patients). Both MP and TPMP increased compared with baseline, with TPMP exceeding MP (median 23 [IQR 20.6–28.5] vs. 22 [19.9–25.6] J/min). The difference between MP and TPMP during CMV/AC was statistically significant (*p* = 0.0016).

In the CPAP/PSV mode, 20 paired measurement sets were available (20 patients). In contrast to CMV/AC, TPMP values during CPAP/PSV were lower than in MP (median 14 [IQR 9.6–18.6] vs. 20 [16.7–26.3] J/min). This difference was statistically significant (*p* < 0.0001).

Under SIMV mode, 20 paired measurement sets were analyzed (20 patients). MP values remained higher than TPMP (median 23 [IQR 17.1–25.9] vs. 17 [14.2–23.5] J/min), and the difference was statistically significant (*p* = 0.0073).

Overall, ventilation mode was significantly associated with the relationship between MP and TPMP (repeated-measures Friedman test, *p* < 0.0001), with TPMP exceeding MP during CMV/AC while remaining consistently lower during CPAP/PSV and SIMV; airway-based MP alone did not differ significantly across conditions in this repeated-measures analysis (Table 2).

When comparing measurements obtained during CMV/AC with those obtained during CPAP/PSV, oxygenation assessed by the PaO_2_/FiO_2_ ratio was numerically higher during CPAP/PSV; however, the difference did not reach statistical significance (*p* = 0.0765). In contrast, respiratory-system compliance was significantly higher during CPAP/PSV (*p* = 0.0002), whereas PEEP (*p* = 0.0241) and plateau pressure (*p* = 0.0066) were significantly higher during CMV/AC. Transpulmonary end-inspiratory pressure was significantly higher during CPAP/PSV (*p* = 0.0003), whereas end-inspiratory esophageal pressure was significantly higher during CMV/AC (*p* = 0.0001). Median and interquartile range values for these measurements are presented in Table 3.

## 4. Discussion

The central finding of our study is that the magnitude and direction of the difference between airway-based MP and TPMP varied according to ventilation mode. During CMV/AC, TPMP was higher than conventional MP, whereas during CPAP/PSV and SIMV, TPMP was lower than conventional MP. These findings indicate that airway-based and transpulmonary pressure-based approaches provide different estimates of mechanical load and that their relationship may be influenced by ventilation mode, chest-wall mechanics, and spontaneous respiratory activity. Because TPMP incorporates esophageal pressure-derived estimates of pleural pressure, it may provide complementary physiological information regarding the pressure applied across the lung that is not captured by airway pressure alone. However, our study does not establish that TPMP is a more accurate measure of lung parenchymal stress or VILI, as neither MP nor TPMP was validated against an independent physiological or clinical reference standard. The interpretation of mechanical power during spontaneous and assisted ventilation requires particular caution. Conventional mechanical power equations were developed primarily to quantify the energy transferred from the ventilator to the respiratory system and therefore rely largely on ventilator-derived variables. During CPAP/PSV and SIMV, however, respiratory muscle activity contributes additional mechanical energy that may not be adequately represented by airway pressure measurements. A vigorous inspiratory effort may generate markedly negative pleural pressure, increasing transpulmonary pressure and potentially regional lung stress despite relatively low airway pressures. Thus, ventilator-derived MP may underestimate the total mechanical load experienced by the lung when substantial spontaneous respiratory effort is present.

The incorporation of esophageal pressure provides additional information regarding pleural pressure changes and therefore helps characterize the transpulmonary pressure generated during spontaneous breathing. Nevertheless, the TPMP calculation used in the present study remains an adaptation of a ventilator-derived mechanical power equation. Substitution of transpulmonary driving pressure for ΔP does not fully quantify respiratory muscle work, nor does it provide a complete estimate of the combined mechanical energy generated by the ventilator and the patient. Accordingly, the lower calculated TPMP observed during CPAP/PSV should not be interpreted as demonstrating that the total mechanical energy or potential injurious load applied to the lung was necessarily lower. Rather, our findings demonstrate that airway-based and transpulmonary pressure-based calculations behave differently under conditions in which spontaneous respiratory effort contributes to respiratory mechanics [19,20,21,22,23].

During CMV/AC, the chest wall is passive and more relaxed, resulting in higher respiratory compliance and lower resistance. However, in extrapulmonary ARDS or cases with high intra-abdominal pressure, chest wall elastance is often elevated [21,22]. Most of the pressure applied by the ventilator is directed to the lung parenchyma. Consequently, TPMP is higher than MP (+7.0%). In our cohort, calculation based on ΔP alone yielded lower mechanical-power estimates than the transpulmonary pressure-based calculation during CMV/AC. This finding suggests that the contribution of chest-wall mechanics may influence the relationship between airway-based and transpulmonary estimates of mechanical load [11]. This aligns with previous reports indicating that ΔP can be misleading in patients with low chest wall compliance, creating a false sense of “lung-protective ventilation” [10].

In comparison, the CPAP/PSV mode demonstrated inverse results. In this breathing mode, respiratory muscle effort creates negative pleural pressure, pulling the chest wall inward [16]. A significant portion of the work involves overcoming chest wall resistance and tissue inertia [5]. During CPAP/PSV, airway-based MP was approximately 30.6% higher than the calculated TPMP. This discrepancy demonstrates that airway-based and transpulmonary pressure-based calculations provide substantially different estimates under spontaneous breathing conditions. However, it should not be interpreted as evidence that airway-based MP overestimates the true lung load by 30.6%, because patient-generated respiratory muscle work is not fully captured by either calculation [20]. This difference may be clinically important; reliance on MP alone, without consideration of TPMP, may result in an inaccurate characterization of the mechanical load [13]. The observed differences between MP and TPMP across ventilation conditions raise the possibility that the two approaches may provide different physiological information during ventilatory management. However, the present study did not evaluate whether these differences influenced clinical decision-making, sedation practices, timing of weaning, or duration of mechanical ventilation. Therefore, no conclusions regarding the clinical benefit of TPMP-guided management can be drawn from these data.

An important consideration when interpreting the differences between CMV/AC and CPAP/PSV is the non-randomized, within-subject nature of the protocol. All four ventilation conditions were assessed sequentially within a single predefined physiological protocol session per patient (Section 2), but the order of conditions was fixed rather than randomized, progressing from deeper to lighter sedation. Consequently, differences in MP and TPMP may have been influenced not only by ventilation mode itself but also by the accompanying, non-randomized change in sedation depth, respiratory drive, and respiratory muscle activity across the fixed condition sequence. Indeed, respiratory system compliance, PEEP, plateau pressure, transpulmonary pressure, and esophageal pressure differed between CMV/AC and CPAP/PSV in our cohort. Therefore, the present data cannot isolate an independent causal effect of ventilation mode from the effect of the accompanying change in sedation depth within the fixed protocol sequence, and the observed differences should be interpreted as associations with ventilation condition rather than as evidence of a causal effect of ventilation mode itself.

MP calculated during SIMV reflected a mix of controlled and spontaneous ventilation, and the divergence between the two indices was again apparent, with TPMP 14.9% lower. Interestingly, MP peaked in this ventilation mode (26.7 J/min), likely reflecting patient–ventilator asynchrony or the patient’s resistance to mechanical ventilation [23]. Clusters of ineffective efforts and asynchronies are known to be associated with poorer outcomes [24].

Clinical implications may vary depending on the mode of ventilation. During controlled ventilation in CMV/AC, MP may appear low, masking a higher TPMP, particularly in ARDS patients with poor lung compliance [11], while during CPAP/PSV ventilation, MP remains relatively high. The observed divergence between MP and TPMP during CPAP/PSV suggests that airway-based MP alone may not fully characterize changes in transpulmonary mechanical load during spontaneous breathing. However, the present study did not evaluate whether these differences affected weaning decisions, sedation management, or clinical outcomes; therefore, their clinical implications remain to be established [25].

Based on our preliminary findings, we hypothesize that esophageal pressure monitoring may add physiological information in moderate-to-severe ARDS patients ventilated in CMV/AC mode with deep sedation and that airway-based MP alone may not adequately characterize transpulmonary load during CPAP/PSV. These hypotheses were not tested against clinical decision-making, VILI, or outcome data in this retrospective study and require confirmation in prospective, adequately powered trials to determine any impact on clinical decision-making or outcomes [12,19]. While MP is a strong predictor of mortality in large cohorts [26], its application in coronavirus and other specific ARDS etiologies continues to evolve [27]. Future prospective trials are needed to validate these findings and identify optimal TPMP thresholds [28]. In our study, we found that while oxygenation did not significantly differ between ventilation modes, CPAP/PSV was associated with significantly better respiratory system compliance and lower ventilatory pressures, whereas CMV/AC required higher PEEP and plateau pressures, reflecting more compromised lung mechanics. CPAP/PSV was also associated with greater inspiratory transpulmonary pressure and lower esophageal pressure, suggesting different patterns of respiratory effort and mechanics between the two ventilation strategies, as demonstrated in Table 3.

Importantly, statistical significance should be distinguished from clinical relevance when interpreting these findings. At baseline, although the difference between MP and TPMP reached statistical significance, the absolute difference was small (approximately 1 J/min), and the distributions substantially overlapped. Therefore, this finding should not be interpreted as evidence of a clinically meaningful difference between the two measures at baseline. In contrast, the magnitude of the discrepancy was greater during CPAP/PSV and SIMV, suggesting that the relationship between airway-based and transpulmonary estimates of mechanical load may become more pronounced as ventilatory conditions and spontaneous respiratory activity change. Nevertheless, the present study was not designed to establish a minimum clinically important difference for MP or TPMP, and no validated threshold currently allows the observed numerical differences to be directly translated into differences in clinical outcomes. Accordingly, the clinical significance of these differences requires confirmation in larger prospective studies.

Our study has several limitations. These include the small sample size (N = 20), retrospective design, and lack of validation of TPMP against long-term clinical outcomes such as mortality. In addition, esophageal pressure is an indirect estimate of pleural pressure and is subject to technical and physiological sources of variability. Given the retrospective nature of the study, complete standardization or retrospective verification of all technical aspects of esophageal pressure measurement may not have been possible. Therefore, the absolute TPMP values and differences between ventilation modes should be interpreted cautiously, and prospective studies using standardized esophageal pressure measurement protocols are needed to confirm our findings. Furthermore, because this is a retrospective observational study, the lower absolute TPMP values observed during CPAP/PSV may be partially attributable to improved underlying respiratory mechanics during spontaneous breathing rather than to the ventilation mode itself, acting as a potential confounding variable when directly comparing absolute power values between modes. Furthermore, ventilation mode was not randomly assigned within the protocol; conditions were assessed in a fixed order that progressed from deeper to lighter sedation. Differences in sedation depth, respiratory drive, and respiratory muscle activity associated with this fixed order were not independently randomized and may have contributed to the observed differences between ventilation conditions. Therefore, causal effects of ventilation mode on MP or TPMP, independent of the accompanying change in sedation depth, cannot be inferred from the present data. An additional limitation concerns the application of ventilator-derived mechanical power equations during spontaneous and assisted ventilation. During CPAP/PSV and SIMV, patient-generated respiratory muscle work contributes to the total mechanical energy applied to the respiratory system and lung but is not fully captured by ventilator-derived variables. Although esophageal pressure allows estimation of pleural and transpulmonary pressure changes, the TPMP equation used in this study does not provide a complete quantification of respiratory muscle work or total patient-plus-ventilator mechanical power. This limitation is particularly relevant to the interpretation of the CPAP/PSV and SIMV findings.

## 5. Conclusions

This exploratory study demonstrates that airway-based MP and esophageal pressure-derived TPMP provide different estimates of mechanical load and that the magnitude and direction of these differences vary across ventilation conditions. These findings support the physiological relevance of considering transpulmonary pressure when characterizing respiratory mechanical load. However, this study does not establish that TPMP-guided ventilation improves clinical outcomes or reduces VILI. The potential clinical value of TPMP should therefore be evaluated in larger prospective studies.

## Figures and Tables

**Table 1:** Patient baseline characteristics, disease severity, and clinical outcomes.

Variable (N = 20)	Value
Age (years), median (IQR)	72.5 (57.8–79.0)
Male sex, n (%)	15 (75%)
Female sex, n (%)	5 (25%)
BMI (kg/m^2^), median (IQR) (n = 18)	24.6 (22.9–27.6)
Hospital length of stay (days), median (IQR)	43.0 (17.2–61.2)
ICU length of stay (days), median (IQR)	14.0 (9.8–29.8)
APACHE II score (points), median (IQR)	25.5 (17.0–27.0)
APACHE II predicted mortality (%), median (IQR)	54.8 (27.1–63.0)
Observed in-hospital mortality, n (%)	10 (50%)
ARDS at intubation, n (%)	18 (90%)
ARDS on ICU admission, n (%)	16 (80%)
ARDS developed during ICU stay, n (%)	6 (30%)
Pneumonia on admission, n (%)	15 (75%)
Ventilator-free days, median (IQR)	1.0 (0.0–4.0)
AKI-free days, median (IQR) (n = 16)	1.5 (0.0–7.5)
RRT-free days, median (IQR) (n = 16)	3.5 (0.0–10.25)
Vasopressor-free days, median (IQR)	5.0 (1.0–11.75)
VAP, n (%)	7 (35%)
Time to VAP onset, median (IQR), days (n = 7)	9.0 (5.5–15.0)
Successfully extubated, n (%)	9 (45%)
Not extubated or died before extubation, n (%)	11 (55%)
Extubation > 48 h, n (%)	8 (40%)
Reintubation after 48 h, n (%)	4 (20%)
Tracheostomy performed, n (%)	6 (30%)

BMI, body mass index; ICU, intensive care unit; AKI, acute kidney injury; ARDS, acute respiratory distress syndrome; RRT, renal replacement therapy; VAP, ventilator-associated pneumonia. All continuous variables are presented as median (IQR), consistent with Section 2.2.

**Table 2:** Mechanical power and transpulmonary mechanical power by ventilation mode.

Ventilation Mode	Mechanical Power Median (N = 20)	Transpulmonary Mechanical Power Median (N = 20)	*p* Value
Baseline	20 (17.1, 20.9)	20 (16.8, 21.8)	*p* = 0.01
CMV/AC	22 (19.9, 25.6)	23 (20.6, 28.5)	*p* = 0.001
CPAP/PSV	20 (16.7, 26.3)	14 (9.6, 18.6)	<0.0001
SIMV	23 (17.1, 25.9)	17 (14.2, 23.5)	0.0073

CMV, controlled mechanical ventilation; CPAP/PSV, continuous positive airway pressure/pressure support ventilation; SIMV, synchronized intermittent mandatory ventilation.

**Table 3:** Respiratory mechanics according to ventilation condition.

Measurements Total n = 20	CMV/AC + Deep Sedation (Median)	CPAP/PSV (Median)	*p* Value
**PaO_2_/FiO_2_ ratio, mmHg**	184 (153.0, 202.2)	194 (155.5, 230.2)	0.0765
**Respiratory system compliance, mL/cm H_2_O**	25 (20.8, 27.6)	35 (30.0, 39.5)	0.0002
**PEEP, cmH_2_O**	15 (13.5, 20.0)	12 (10.0, 15.0)	0.0241
**Plateau pressure, cmH_2_O**	31 (30.7, 33.6)	26 (22.8, 28.6)	0.0066
**Transpulmonary end-inspiratory pressure, cm H_2_O**	12 (10.7, 14.4)	25 (20.5, 30.5)	0.0003
**Esophageal end-inspiratory pressure, cm H_2_O**	21 (17.6, 22.4)	4 (−0.9, 7.4)	0.0001

CMV/AC, controlled mechanical ventilation/assist-control; CPAP/PSV, continuous positive airway pressure/pressure support ventilation; PEEP, positive end-expiratory pressure.

## Data Availability

All data generated or analyzed during this study are included in this published article. The datasets used and/or analyzed during the current study are available from the corresponding author upon reasonable request.

## Supplementary Materials

The following supporting information can be downloaded at: https://www.mdpi.com/article/10.3390/life16101662/s1, Table S1: Ventilator settings and sedation depth by ventilation condition.
