# Supplementary material for: Comparison of Airway-Based and Transpulmonary Mechanical Power Across Ventilation Conditions in Moderate-to-Severe ARDS
Source: Life (Basel). 2026 Oct 3;16(10):1662. doi: 10.3390/life16101662 (PMC13706505; doi:10.3390/life16101662)
Supplement: Supplementary file 1 [file life-16-01662-s001.zip › life-4406255-supplementary.pdf]

Supplementary Table S1. Ventilator settings and sedation depth by ventilation condition

Variable	Baseline (n=20)	CMV/AC + deep sedation (n=20)	SIMV + light sedation (n=20)	CPAP/PSV (n=20)
RASS score, median (IQR)	-4.0 (-5.0, -4.0)	-4.0 (-5.0, -4.0)	-2.0 (-2.0, -2.0)	-1.0 (-1.2, -1.0)
FiO2, %, median (IQR)	67 (60, 70)	60 (60, 66)	50 (45, 60)	50 (49, 55)
Set inspiratory pressure / pressure-support level above PEEP, cmH2O, median (IQR)	19.3 (17.5, 20.0)	19.0 (17.5, 20.0)	16.0 (15.0, 18.0)	16.0 (13.8, 18.0)
Peak inspiratory pressure (PIP), cmH2O, median (IQR)	30.0 (28.8, 31.0)	34.5 (32.0, 37.0)	29.6 (28.0, 31.0)	28.0 (25.7, 31.0)
Plateau pressure, cmH2O, median (IQR)	27.5 (25.4, 28.2)	31.3 (30.7, 33.6)	26.5 (24.9, 28.1)	26.0 (22.8, 28.6)
PEEP, cmH2O, median (IQR)	11.0 (10.0, 12.0)	15.0 (13.5, 20.0)	11.0 (10.0, 15.2)	12.0 (10.0, 15.0)
Tidal volume, mL, median (IQR)	444 (360, 505)	450 (400, 485)	500 (465, 555)	550 (490, 580)
Respiratory rate, breaths/min, median (IQR)	20.5 (17.8, 24.0)	20.0 (18.0, 24.0)	20.0 (16.0, 22.0)	18.0 (16.0, 22.5)

RASS, Richmond Agitation-Sedation Scale (more negative = deeper sedation); FiO_2, fraction of inspired oxygen; PEEP, positive end-expiratory pressure. During CPAP/PSV the recorded respiratory rate is entirely patient-triggered (spontaneous); during SIMV it reflects a mixture of mandatory and patient-triggered breaths; during CMV/AC it reflects the set controlled rate under deep sedation. Values are median (IQRs), derived from the study's source ventilator/blood-gas data sheets.
